# Explicit dynamics analysis of forearm tendon stresses during the forehand smash

**DOI:** 10.3389/fbioe.2026.1780880

**Published:** 2026-03-18

**Authors:** Xiaoge Xiao, Jiahua Li, Ao Lian, Wenbin Li, Xuanyi Ou, Zhengyi Lu, Yifang Fan

**Affiliations:** 1 Foot Research Laboratory, School of Physical Education and Sport Science, Fujian Normal University, Fuzhou, China; 2 School of Physical Education and Sport Science, Xi’an Jiaotong University, Xi’an, China

**Keywords:** badminton, elbow injury, finite element, forehand smash, inverse dynamics

## Abstract

**Introduction:**

Fast, asymmetric strokes in racket sports generate complex multi-joints loads that affect performance and injury; however, the biomechanical mechanisms of milliseconds-scale shuttlecock-racket collisions are not fully elucidated. This paper aims to characterize the contact kinetics of the badminton forehand smash and evaluate the resulting stress and strain distribution within the forearm during the impact contact.

**Methods:**

Seven badminton athletes were recruited, and forehand smash trials were captured with a motion-capture system at 1,000Hz. Using inverse dynamics, we computed the sweep angular velocities, centripetal acceleration and centrifugal forces of racket, hand and forearm relative to the shoulder. Further, we reconstructed a subject-specific upper-limb model comprising the bone, soft tissues, and the racket. Kinetics inputs were applied as loading boundary conditions to an explicit dynamics finite element solver to obtain von Mises equivalent stress and strain for six tendons: flexor digitorum superficialis, flexor carpi ulnaris, pronator teres, extensor digitorum communis, extensor carpi ulnaris, and extensor carpi radialis brevis. Kinetics and finite element results were defined with a normalized contact window (0%–100%).

**Results:**

The shuttlecock-racket contact window was approximately 3 ms. Racket kinematics peaked one frame at the impact and declined during contact. Shuttlecock speed surged early, with anterior-posterior-dominant impact force peaking in the first 1 ms. Hand sweep angular velocity was approximately constant while forearm sweep angular velocity rose slightly then fell. Furthermore, extensor digitorum communis exhibited the highest stress, followed by the flexor digitorum superficialis and pronator teres, with the extensor carpi radialis brevis lowest. Flexor digitorum superficialis displayed the largest strain. In terms of timing, stresses in pronator teres and flexor digitorum superficialis rose rapidly from 0% to 30% of the contact window; from 30% to 70%, pronator teres reached its peak, with extensor digitorum communis peaking in the same interval.

**Discussion:**

Impulse transmission in the forehand smash is driven by forearm pronation and wrist flexor–extensor co-contraction to stiffen the distal segments. The extensor digitorum communis and pronator teres carry relatively higher loads, supporting eccentric strengthening of the wrist extensors and the pronation chain to reduce cumulative overuse risk under high-repetition loading.

## 1 Introduction

Racket sports are widely promoted for their health benefits (Oja et al., 2017). Badminton is the fastest of these sports; during a forehand smash, peak racket-head speed can exceed 111 m/s (Ramasamy et al., 2024). The forehand smash is the most common stroke in badminton and follows a proximal-to-distal sequential kinetic chain: angular momentum generated by the shoulder and trunk is transferred distally, producing whiplike acceleration of the racket and wrist and thereby increasing shuttlecock speed (Wagner et al., 2014). Similar mechanisms are observed in tennis, squash and baseball (Nhan et al., 2018). Previous work in biomechanics of forehand smash in badminton has focused on key performance determinants: larger shoulder internal-rotation angles are associated with higher shuttlecock speed (King et al., 2020), and whole-body kinetics models indicate that elbow and wrist flexion-extension capacity significantly influences smash speed (Zhang et al., 2016). However, during acceleration and deceleration–particularly in the rapid eccentric-braking phase–the distal segments of the kinetic chain (elbow, forearm, wrist, and hand) undergo very high angular velocities and inertial loads, producing localized tendon and soft-tissue stress concentrations that increase the risk of eccentric injury (Beaudry et al., 2022). Epidemiological evidence indicates that ∼20% of badminton players sustain upper-limb injuries, of which elbow tendinopathies account for ∼70% (Shariff et al., 2009), and they are also prevalent in other overhead racket sports and in occupations requiring repetitive forearm rotation (Nhan et al., 2018; Rosa et al., 2019). However, current research on upper-limb injuries has focused on epidemiology or on muscle strength balance (Couppé et al., 2014; Pardiwala et al., 2020), and there is an urgent need for mechanistic analyses.

Due to the complex interactions caused by the rapid contact between the shuttlecock and the racket in badminton, higher sampling rates are required for motion capture systems. Typical motion-capture rates in badminton range from 200 to 700 Hz, with the highest reported rate to date being 700 Hz (Tsai et al., 2000; Kwan and Rasmussen, 2010; Ramasamy et al., 2024). Accurate data acquisition is a prerequisite for reliable results. According to the Nyquist-Shannon sampling theorem, a signal must be sampled at no less than twice its highest frequency component (Shannon, 1998; Song and Godøy, 2016). Current research shows that shuttlecock-racket contact during a forehand smash lasts only approximately 4 m (Tsai et al., 2000), necessitating higher temporal resolution to resolve this interaction.

Conventional models can estimate joint-level forces and moments, but they have limited ability to capture microstructural deformation, stress distributions and tissue interactions within tendons, cartilage and ligaments (Hedenstierna et al., 2008; Ramasamy et al., 2024; Tajik et al., 2025). The finite element method is well suited to solving strongly nonlinear boundary-value problems across temporal and spatial scales (Yu et al., 2020), and has been used to evaluate tissue-level loads and injury risk in the musculoskeletal system (Islán Marcos et al., 2019). When combined with *in vivo* and *ex vivo* data, finite element method can improve understanding of injury mechanisms (Wren et al., 2003; Yu et al., 2020). Finite element analyses of racquet-sport implements (e.g., badminton and tennis rackets) have been reported (Goodwill et al., 2005; Suwannachote et al., 2023), and related investigations into upper-limb injuries are increasingly common. Simulations of tennis strokes across different elbow flexion angles indicate that using a smaller elbow flexion angle can reduce the risk of radial injury (Chen et al., 2023). Another study showed that when the impact location or direction deviates from the central axis of the upper arm, the stress borne by the humerus during baseball batting decreases (Sakai et al., 2008). Moreover, during the deceleration phase of throwing, peak stress of approximately 109.6 MPa can occur at the origin of the biceps tendon, indicating a risk of soft-tissue tearing (Yeh et al., 2005). By comparison, finite element studies in badminton have focused largely on the lower limb, and investigations of the upper limb remain scarce (Lam et al., 2020). Meanwhile, static analyses struggle to represent the transient characteristics of quasi-collision events such as rapid racket swings; by contrast, explicit dynamics can accurately capture nonlinear contact and inertial effects, yielding a more realistic contact-force time history, impulse transmission, and energy dissipation (Suwannachote et al., 2023).

To address these limitations, we developed an anatomically realistic, coupled racket-upper-limb model. We synchronously captured the kinetics of the shuttlecock, racket and upper limb at 1,000 Hz and, via inverse dynamics, computed shuttlecock-racket contact window and segmental centrifugal forces, which were then used as loading inputs for explicit dynamics analyses. As an initial step, this integrated framework has the potential to characterize tissue level stress-strain and injury risk during badminton forehand smash, potentially offering mechanical insights to inform injury prevention and evidence-based training.

## 2 Methods

### 2.1 Participants

We recruited seven male badminton athletes with no history of neuromuscular disorders or major disease and in good health. They all had participated in international competitions. All participants refrained from vigorous training or competition for at least 48 h before testing. In the post-test analysis, the participant who best matched the study objective and achieved the highest forehand smash speed was selected to undergo computed tomography. The study was approved by the Ethics Committee of Fujian Normal University (approval no. 2021001). All participants were informed about the study procedures and provided written informed consent prior to participation. Data collection took place on 11–13 May 2021.

### 2.2 Equipment

A Qualisys motion-capture system used comprising 18 cameras (16 Oqus 700+ infrared motion-capture cameras and 2 Oqus 210c color high-speed video cameras), a calibration wand, a calibration L-frame, and Qualisys Track Manager software (QTM, v2.15). The badminton racket was a Li-Ning FLAME N65 (mass, 100.0 g). The shuttlecock was a Li-Ning A+300 (mass, 5.0 g), which weighed 5.6 g after reflective tape was applied.

### 2.3 Testing protocol

**FIGURE 1:**
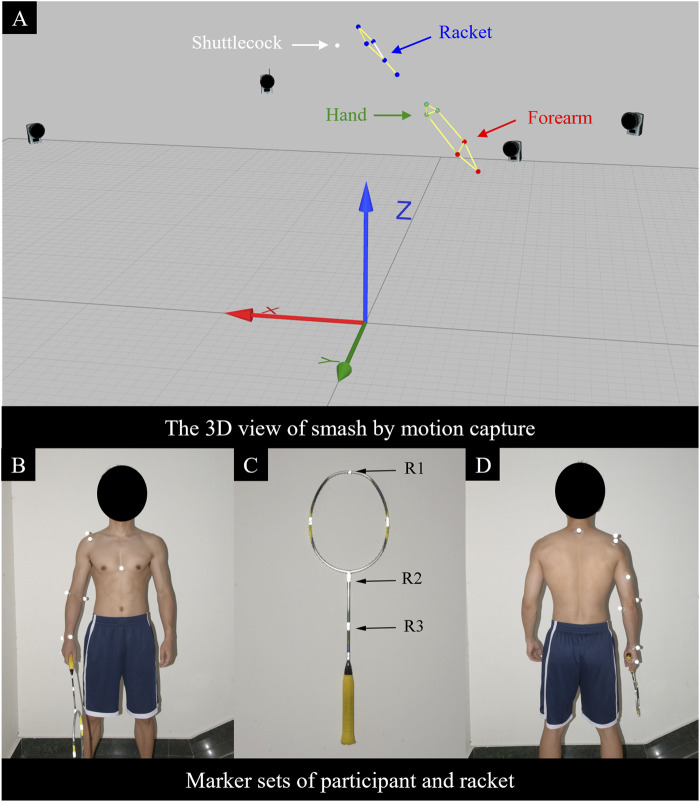
Motion-capture volume and marker sets protocol. **(A)** Forehand smash sequence displayed in Qualisys software. Laboratory coordinate system: x, anterior-posterior; y, medial-lateral; z, superior-inferior. **(B)** Frontal view of participant marker placement. **(C)** Racket marker placement. **(D)** Posterior view of participant marker placement.

Participants completed a standardized warm-up. Reflective markers were placed on anatomical landmarks according to the Qualisys Animation Marker Set, and reflective tape was affixed at the junction of the racket head and shaft. Sixteen infrared motion-capture cameras were arranged circumferentially around the court to provide 360° coverage, and two color high-speed video cameras were positioned anteriorly and posteriorly. Camera heights and tilt angles were adjusted to the participant’s motion to ensure complete capture (Figure 1). The sampling rate was set to 1,000 Hz and the exposure time to 0.1 m. Forehand smash trials were collected, with three attempts per participant. Participants were afforded ample rest, and the interval between attempts was self-paced. After each acquisition, shuttlecock reflective-marker trajectories were processed in Qualisys Track Manager using a zero-lag, fourth-order Butterworth low-pass filter with a 40 Hz cut-off (Winter 2009; Morrison et al., 2018). For each participant, the forehand smash with the highest shuttlecock speed among the three trials was retained for analysis (Fleisig et al., 1995).

### 2.4 Kinetics modeling analysis

We constructed a subject-specific skeletal model in Qualisys Track Manager and, based on this model, exported kinematic variables for the shuttlecock, racket, and key upper-limb joints (wrist, elbow, shoulder), including shuttlecock speed, centripetal acceleration, and sweep angular velocity. To account for inter-individual variability in inertial properties arising from differences in bone, muscle, and adipose tissue composition (Rao et al., 2006), segment masses and moments of inertia for the upper limb were estimated using established regression equations and scaled to individual anthropometrics, enabling subject-specific modeling and comparable dynamic solutions. The rationale for addressing body-composition differences via regression-based estimation follows prior work (Yeadon and Morlock, 1989).

We defined the capture volume in the positive octant of a right-handed Cartesian frame (*x, y, z*). Let the frame interval be Δ*t* (sampling frequency $f_{s}$ = 1/Δ*t*). The shuttlecock position at time *t* is $p\left(t\right)=\left[x\left(t\right),y\left(t\right),z\left(t\right)\right]$ in the global frame. Displacement trajectories $x\left(t\right)$ , $y\left(t\right)$ , and $z\left(t\right)$ were filtered prior to numerical differentiation using a 4th-order low-pass Butterworth filter with a 40 Hz cutoff frequency. No additional filtering was applied to the derived velocity (Equations 1–3), which were computed from the filtered displacement as

$$v_{x}\left(t\right)=\frac{dx\left(t\right)}{dt}$$ (1)

$$v_{y}\left(t\right)=\frac{dy\left(t\right)}{dt}$$ (2)

$$v_{z}\left(t\right)=\frac{dz\left(t\right)}{dt}$$ (3)

Acceleration components (Equations 4–6) were then obtained as the second time derivatives of the filtered displacement

$$a_{x}\left(t\right)=\frac{d^{2}x\left(t\right)}{dt^{2}}$$ (4)

$$a_{y}\left(t\right)=\frac{d^{2}y\left(t\right)}{dt^{2}}$$ (5)

$$a_{z}\left(t\right)=\frac{d^{2}z\left(t\right)}{dt^{2}}$$ (6)

Resultant speed (Equation 7) and acceleration magnitudes (Equation 8) are

$$v=\sqrt{{v_{x}\left(t\right)}^{2}+{v_{y}\left(t\right)}^{2}+{v_{z}\left(t\right)}^{2}}$$ (7)

$$a=\sqrt{{a_{x}\left(t\right)}^{2}+{a_{y}\left(t\right)}^{2}+{a_{z}\left(t\right)}^{2}}$$ (8)

Within the shuttlecock-racket contact window, the force components (Equations 9–11) acting on the shuttlecock of mass m were estimated by

$$F_{x}=ma_{x}\left(t\right)$$ (9)

$$F_{y}=ma_{y}\left(t\right)$$ (10)

$$F_{z}=ma_{z}\left(t\right)$$ (11)

The resultant force magnitude (Equation 12), defined as the Euclidean norm of the force vector F $\left(t\right)=\left[F_{x}\left(t\right),F_{y}\left(t\right),F_{z}\left(t\right)\right]$

$$F\left(t\right)=\sqrt{{F_{x}\left(t\right)}^{2}+{F_{y}\left(t\right)}^{2}+{F_{z}\left(t\right)}^{2}}=m\sqrt{{a_{x}\left(t\right)}^{2}+{a_{y}\left(t\right)}^{2}+{a_{z}\left(t\right)}^{2}}$$ (12)

Taking the shoulder joint as the rotation center *O,* we defined position vectors for the racket center of mass, wrist, and elbow relative to *O* (Equation 13)

$$r_{k}\left(t\right)\in \left\{\text{racket},\text{hand},\text{forearm}\right\}$$ (13)

The frame-to-frame change is (Equation 14)

$$\Delta r_{k}=r_{k}\left(t+\Delta t\right)-r_{k}\left(t\right)$$ (14)

and the instantaneous rotation (Equation 15) radius is the Euclidean norm

$$r_{k}=\left\|r_{k}\left(t\right)\right\|$$ (15)

Using the law of cosines on the triangle formed by $r_{k}\left(t\right)$ , $r_{k}\left(t+\Delta t\right),$ and chord $\left\|\Delta r_{k}\right\|$ , the inter-frame angular displacement (Equation 16) is

$$\theta_{k}\left(t\right)=\arccos\left(\frac{r_{k}^{2}\left(t\right)+r_{k}^{2}\left(t+\Delta t\right)-{\left\|\Delta r_{k}\right\|}^{2}}{2r_{k}\left(t\right)r_{k}\left(t+\Delta t\right)}\right)$$ (16)

and the sweep angular velocity (Equation 17) is

$$\omega_{k}=\frac{\theta_{k}}{\Delta t}$$ (17)

For the racket, hand, and forearm (respective masses $m_{k}$ ), the centrifugal force (Equation 18) about the shoulder was estimated as

$$F_{cf,k}=m_{k}\omega_{k}^{2}r_{k},k\in \left\{\text{racket},\text{hand},\text{forearm}\right\}$$ (18)

We focused on the shuttlecock–racket contact window. The contact window was identified from kinematic trajectory data, after which the time axis within this window was linearly mapped to 0%–100% and uniformly resampled to 100 normalized points using Python (PyCharm). To suppress sampling and numerical noise, the normalized sequences were denoised and smoothed in Origin via low-order polynomial fitting, with the polynomial order held constant across analyses. Percentile curve was then obtained by pointwise aggregation across participants and reported at each normalized time point as mean ± SD. Unless otherwise stated time normalization is applied only within the contact window.

### 2.5 Finite element analysis

#### 2.5.1 Geometric model reconstruction

Participants’ upper limbs were scanned using computed tomography, participants were instructed to maintain the forehand smash posture (i.e., the arm angle/configuration at impact) to ensure the fidelity of the model used in subsequent simulations and the tomographic images were imported into Mimics (Mimics Research 17.0 for x64; Materialise, Leuven, Belgium) for editing, segmentation, and region filling. Based on the open-source Z-Anatomy platform (Z-Anatomy; open-source, Montpellier, France), we constructed detailed anatomical models of the flexor carpi ulnaris, flexor digitorum superficialis, pronator teres, extensor digitorum communis, extensor carpi ulnaris, and extensor carpi radialis brevis. The brachioradialis, pronator teres, flexor carpi radialis, palmaris longus, flexor digitorum superficialis, and flexor carpi ulnaris play indispensable roles in forearm pronation-supination (Ljung et al., 1999; Frederic, 2005). Notably, owing to the low prevalence of palmaris longus agenesis and its functional redundancy, the absence of palmaris longus is typically neglected in clinical and research settings (Sebastin et al., 2005).

Following 3D reconstruction of the right upper limb, we standardized its local anatomical coordinate system to ensure consistency between the coordinate frames used for motion capture and simulation analyses (*x*: anterior-posterior; *y*: medial-lateral; *z*: superior-inferior) (Yu et al., 2020). The surface model was generated in Geomagic (Geomagic Studio 12.0; Geomagic Inc., North Carolina, United States), and components were assembled in SolidWorks (SolidWorks 2016; Dassault Systemes Inc., Massachusetts, United States). A badminton racket geometry was created based on the Li-Ning FLAME N65 (Li-Ning, China). In the finite-element environment, ligaments were represented using spring elements (Peng et al., 2022). These steps yielded a finite-element geometric model comprising the racket, bone, cartilage, ligaments, and muscles.

#### 2.5.2 Material properties and boundary conditions

All materials were assumed to be isotropic and linear elastic materials, and their properties were obtained from previous studies. The shuttlecock was modeled as sphere solid to avoid instability during simulation. The detailed mesh parameters and material properties were presented in Table 1 (Allen et al., 2009; Li et al., 2009; Islán Marcos et al., 2019; Wang et al., 2021).

**TABLE 1:** Material properties of the finite element model.

Properties	Bone	Tendon	Muscle	Cartilage	Racket	String bed	Shuttlecock
Density (g/cm^-3^)	1.5	1.35	1.35	1.15	1.75	1.1	0.7
Young’s modulus (MPa)	15,000	2000	0.3	8	25,000	7,200	30
Poisson	0.3	0.43	0.43	0.49	0.3	0.3	0.05
Nodes	34,878	8,665	29,973	1,046	14,480	30,874	1,501
Elements	159,269	53,679	128,624	2,770	61,183	51,145	1,280
Element type	Tetrahedron	Tetrahedron	Tetrahedron	Tetrahedron	Tetrahedron	Tetrahedron	Hexahedron

In order to simulate the smash and capture the mechanical response of the muscles. Finite element analysis was performed in ANSYS Workbench 2024 LS-DYNA explicit dynamics solver, analyze the coupling interaction during the process of smash. In the simulation, the contact between the shuttlecock and the string surface is defined as frictional, all other contacts (including the humerus and the forearm, the forearm and the racket, and the racket and the string) are defined as bonded contact to ensure structural continuity. The friction coefficient of frictional contact is set at 0.2 to simulate the energy loss and sliding effect during actual stroking (Suwannachote et al., 2023). To accurately reproduce the dynamic characteristics of the forehand smash, the load parameters are based on the experimental data obtained by the motion capture system, including the sweep angular velocity, the centrifugal force at the moment of forehand smash (for details, see Table 2 and 3). Corresponding time step control and constraints were imposed on the finite element model through the time series to ensure the stability and accuracy of the calculation. Since the duration of the smash is extremely short, the shuttlecock’s downward displacement can be neglected. Thus, in the simulation, the shuttlecock was assumed to be initially stationary to simplify the model while maintaining computational accuracy and stability. Given the millisecond contact duration, tissues were modeled as isotropic linear elastic materials; nonlinear and viscoelastic effects were not included due to unavailable subject-specific parameters and to maintain numerical stability in explicit dynamics. von Mises equivalent stress and von Mises equivalent strain of six muscles (The connection interface between the humerus and the tendon) within the contact window (including maxima and minima) were inputted. Time-series data were normalized to 100 points (0%–100%), then denoised and smoothed in Origin using a second-order polynomial fitting, with the polynomial order held constant across analyses. Peak values reported were extracted from the raw (unsmoothed) data; the quadratic smoothing was applied for visualization only.

**TABLE 2:** Frame-resolved kinematics of the racket–hand–forearm system.

Timeline	Speed	Sweep angular velocity			Centripetal acceleration		
/	Shuttlecock	Racket	Hand	Forearm	Racket	Hand	Forearm
0	7.85 ± 3.86	41.08 ± 2.14	21.18 ± 2.77	19.68 ± 2.54	1,383.65 ± 145.45	263.66 ± 29.54	155.98 ± 54.50
1	56.37 ± 13.27	50.03 ± 2.89	27.66 ± 2.53	25.59 ± 3.56	2,059.19 ± 171.83	437.67 ± 46.34	262.08 ± 37.57
2	80.69 ± 10.69	36.58 ± 2.34	21.96 ± 2.11	21.00 ± 2.66	1,109.50 ± 170.48	274.36 ± 41.75	175.45 ± 43.59
3	84.89 ± 7.48	35.56 ± 2.94	22.24 ± 2.30	21.64 ± 2.85	1,052.06 ± 133.42	281.81 ± 32.24	185.29 ± 42.47
4	79.56 ± 8.59	33.76 ± 2.81	21.81 ± 2.17	20.65 ± 2.48	944.40 ± 107.83	271.23 ± 45.57	168.64 ± 41.77

Shuttlecock speed, m/s; sweep angular velocity, rad/s; centripetal acceleration, m/s^2^. Frame 0 denotes the pre-impact condition; Frame 1, the instant of racket–shuttlecock contact; and Frame 4, the moment the shuttlecock leaves the racket.

**TABLE 3:** Frame-resolved kinetics of the racket–hand–forearm system.

Timeline	Net force	Impact force			Centripetal force		
/	/	x	y	z	Racket	Hand	Forearm
0	​	​	​	​	−142.31 ± 14.22	−155.28 ± 15.41	−236.41 ± 78.10
1	288.82 ± 68.48	275.28 ± 77.59	−9.37 ± 23.59	−69.80 ± 30.45	−211.08 ± 14.25	−256.52 ± 21.81	−395.81 ± 82.42
2	217.57 ± 72.41	209.64 ± 69.87	2.71 ± 29.48	−49.24 ± 27.35	−112.30 ± 15.98	−160.43 ± 16.63	−274.23 ± 76.79
3	33.74 ± 25.44	16.31 ± 38.94	−1.02 ± 15.41	−4.90 ± 15.26	−106.82 ± 16.36	−163.00 ± 20.50	−283.26 ± 70.48
4	​	​	​	​	−96.60 ± 17.49	−156.67 ± 22.46	−257.79 ± 72.20

Impact force and centrifugal force, N. Frame 0 denotes the pre-impact condition; Frame 1, the instant of racket–shuttlecock contact; and Frame 4, the moment the shuttlecock leaves the racket.

#### 2.5.3 Model validation

To validate the model, we randomly selected one forehand smash trial from a randomly chosen participant. Following the aforementioned inverse dynamics procedure, centrifugal components and shuttlecock-racket impact loads were reconstructed from the trial’s kinematic data and applied to the finite element model. Three racket landmarks–R1 (uppermost edge of the string bed), R2 (string bed-shaft junction), and R3 (mid-shaft) – were exported from QTM as three-dimensional displacement coordinates (*x*, *y*, *z*). The corresponding trajectories on a proportionally scaled finite element racket model were compared frame by frame with the experimental trajectories to assess agreement between simulation and measurement and thereby confirm model validity. Agreement was quantified using mean absolute error and root-mean-square error (RMSE) across the nine landmark–direction components. Absolute displacement errors were computed for three racket markers (R1–R3) in three directions (*x*, *y*, *z*), yielding nine marker–direction components. Overall mean absolute error and RMSE were calculated by aggregating these nine errors, and direction-specific RMSEs were obtained by pooling the three marker errors within each axis (*x*, *y*, and *z*). Definitions of R1-R3 are shown in Figure 1, and validation results are reported in Table 4.

**TABLE 4:** Racket displacement validation results (finite element analysis vs. motion capture).

​	R1			R2			R3		
Components	MC	FEA	Error	MC	FEA	Error	MC	FEA	Error
*x*	156.50	90.49	66.02	109.70	73.13	36.57	83.99	70.81	13.18
*y*	−8.99	−5.53	3.46	3.08	−6.66	9.74	5.57	−5.39	10.97
*z*	−64.03	−43.50	20.53	−52.50	−32.86	19.64	−45.48	−31.47	14.01

The error is expressed as the absolute value of the displacement obtained from motion capture minus that from the finite element results. In both methods, the displacement represents the racket motion within the racket–shuttlecock contact window and is reported in mm. MC, denotes motion capture, and FEA, denotes finite element analysis.

## 3 Results

### 3.1 Kinematic and kinetic results

Mean values of shuttlecock speed, impact force, sweep angular velocity, centripetal acceleration, and centrifugal force during the forehand smash were computed across seven participants; the results are presented in Table 2 and 3. At frame 0, before-impact, the shuttlecock’s speed was approximately 7.85 m/s–close to free-fall.

At frame 1, post-impact, the racket’s sweep angular velocity, centripetal acceleration, and centrifugal force all reached their peak values and began to decline from this frame. The shuttlecock’s acceleration is approximately 48,520 m/s^2^ (frame 0–1). The shuttlecock’s net impact force peaked (288.82 ± 68.48 N), moreover, the anterior-posterior impact component was the largest of the three directions (275.28 ± 77.59 N).

Between frames 2-3, the shuttlecock’s speed continued to rise while the impact force decreased. Over the same frames, the racket’s sweep angular velocity, centripetal acceleration, and centripetal force all showed a downward trend, whereas all corresponding measures for the forearm increased. The hand’s sweep angular velocity, centripetal acceleration, and centripetal force remained largely stable.

At frame 4, the shuttlecock was not in contact with the racket (shuttlecock speed decreased). All measured variables exhibited a downward trend.

**FIGURE 2:**
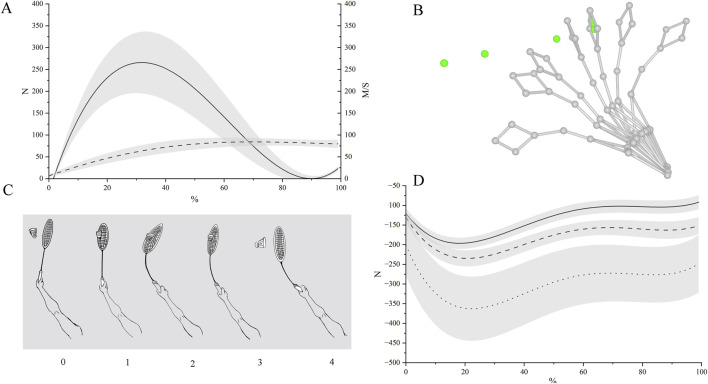
Forehand smash schematic and kinematic-dynamic results. **(A)** Time histories of shuttlecock speed (dashed line) and impact force on the shuttlecock (solid line). **(B)** Skeletal visualization of the forehand smash in Qualisys. **(C)** Phase schematic of the hitting sequence: 0, pre-impact; 1, initial shuttlecock-racket contact; 2, coupled motion of shuttlecock and racket; 3-4, shuttlecock separation. **(D)** Centrifugal force curves during the forehand smash: solid line, racket; dashed line, hand; dotted line, forearm.

Overall, shuttlecock-racket contact lasted 3 m (frames 1–3). After impact the shuttlecock’s speed increased rapidly and impact force peaked at frame 1 before declining through frames 2–3. In parallel, the racket’s sweep angular velocity, centripetal acceleration, and centrifugal force reached their maxima at frame 1 and then progressively decreased through frame 4. During frames 2–3, forearm kinetics increased while hand kinetics remained relatively stable. After separation at frame 4, shuttlecock speed dropped and all measured variables showed a downward trend. See Figure 2C for details.

### 3.2 Finite element results

**FIGURE 3:**
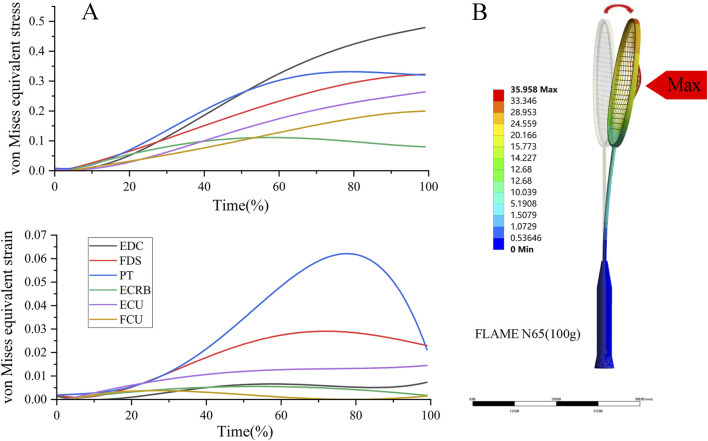
Finite element results. **(A)** Time series of stress and strain within the contact window **(B)** schematic illustration of the racket simulation results. Units: von Mises equivalent stress, MPa, Equivalent strain, dimensionless. EDC, extensor digitorum communis; FDS, flexor digitorum superficialis; PT, pronator teres; ECRB, extensor carpi radialis brevis; FCU, flexor carpi ulnaris; ECU, extensor carpi ulnaris.

Racket displacement predicted by the finite element analysis showed the same overall trend as the motion-capture measurements but with smaller amplitudes in the *x* and *z* directions for all three markers (R1-R3). The absolute errors between methods ranged from 3.46 to 66.02 mm (Table 4). The largest discrepancy occurred at R1 in the *x*-direction (motion capture: 156.50 mm vs. finite element analysis: 90.49 mm; absolute error: 66.02 mm), whereas absolute errors in the *y* direction were relatively small, with the minimum error observed at R1 (3.46 mm). For R2 and R3, the differences between motion capture and finite element remained below 36.57 mm in the *x* direction and below 20.53 mm in the *z* direction. Overall, the mean absolute error was 21.57 mm and the RMSE was 28.09 mm, with direction-specific RMSEs of 44.19 mm (*x*), 8.70 mm (*y*), and 18.28 mm (*z*) (Table 4). Using the kinematic results in Tables 2, 3, we applied the corresponding loading and boundary conditions to the finite element model and solved it. See Table 5 and Figure 3 for the results. Table 5 show that among the six tendons, the extensor digitorum communis exhibited the highest von Mises equivalent stress, followed by the flexor digitorum superficialis and the pronator teres, whereas the extensor carpi radialis brevis showed the lowest values. Further, as shown in Figure 3, within the normalized contact window (0%–100%), von Mises equivalent stresses in the pronator teres and the flexor digitorum superficialis rose rapidly during the early phase (0%–30%). In the mid phase (30%–70%), the pronator teres reached its peak, and the extensor digitorum communis also attained its peak. During the late phase (70%–100%), stresses in the pronator teres, extensor digitorum communis, and flexor digitorum superficialis declined progressively.

**TABLE 5:** Finite element results of six tendons.

​	von mises equivalent stress			von mises equivalent strain		
​	Min	Avg	Max	Min	Avg	Max
Extensor digitorum communis	0.45	0.63	0.75	1.17E-03	1.38E-03	1.50E-03
Flexor digitorum superficialis	0.30	0.37	0.48	1.34E-03	1.65E-03	1.89E-03
Pronator teres	0.27	0.33	0.50	7.11E-04	1.11E-03	1.56E-03
Extensor carpi radialis brevis	0.07	0.09	0.11	1.69E-05	3.01E-05	2.37E-05
Flexor carpi ulnaris	0.24	0.26	0.35	1.12E-03	1.28E-03	1.57E-03
Extensor carpi ulnaris	0.18	0.21	0.30	4.19E-04	5.01E-04	6.66E-04

von Mises equivalent stress, MPa, Equivalent strain, dimensionless.

## 4 Discussion

Using a 1,000 Hz motion-capture system, we recorded forehand mash kinetics in seven professional badminton players and resolved millisecond-scale shuttlecock-racket contact windows. Inverse dynamics yielded angular velocities, centripetal accelerations, and centrifugal forces for the racket, hand, and forearm, revealing a contact duration of approximately 3 m; racket variables peaked at the impact frame (frame 1) and then decreased rapidly after contact began, whereas shuttlecock speed rose steeply early in contact (frames 1–2). Within the contact window, hand sweep angular velocity and centripetal acceleration showed a slight rebound immediately after impact and then remained approximately stable. The validation results showed that the differences between the racket displacement predicted by the finite element model and that measured by motion capture ranged from 3.46 mm to 66.02 mm. Explicit dynamics finite element analysis further indicated that distal stiffening of the distal segments is achieved via engagement of the pronator teres and co-contraction of the wrist flexor/extensor groups: the flexor digitorum superficialis carried relatively high loads, the extensor digitorum communis contributed to distal stabilization, and the pronator teres rose rapidly from contact onset, peaking at ∼60–75% of the contact window to counter reverse impact forces and facilitate efficient impulse transmission.

In theory, the inter-frame interval in motion capture should be short enough to avoid artifact in marker trajectories. According to the Shannon-Nyquist sampling theorem, the sampling frequency must exceed twice the highest frequency component of the motion (Shannon, 1998; Song and Godøy, 2016). In current badminton research, sampling rates commonly range from 200 to 700 Hz (Tsai et al., 2000; Jaitner and Gawin, 2010; Ramasamy et al., 2024). One study reported a 4 m shuttlecock–racket contact duration during a forehand smash (500 Hz sampling; shuttlecock speed 68 m/s) (Tsai et al., 2000), which essentially satisfies the lower bound of the Shannon criterion; however, with only two samples in the contact window, the dynamics at the shuttlecock-racket interface are difficult to reconstruct (Song and Godøy, 2016). By increasing the sampling rate to 1,000 Hz, our study captured four samples within the contact window to enhance data reliability; as shown in Table 2, shuttlecock-racket contact lasted 3 m (frames 1–3). Over these 3 m, shuttlecock speed increased progressively, particularly in early contact (frames 1–2). Using a 700 Hz sampling rate, Ramasamy’s group reported maximum shuttlecock speeds of 97 m/s and 111 m/s (Ramasamy et al., 2024; 2025), broadly consistent with our results in terms of shuttlecock speed and participant levels.

In current research on upper-limb kinetics, the forehand smash has been widely examined. Most existing studies reconstruct joint motion from optical markers to compute joint angles and angular velocities, focusing primarily on these state variables themselves (Rusdiana, 2021). In contrast, our study defines the angular velocities of three segments uniformly as sweep angular velocities relative to the shoulder; consequently, the magnitudes are not directly comparable with reports based on relative joint angular velocities, though the temporal features of the kinetic chain remain comparable (Ramasamy et al., 2023). As shown in Tables 2, 3, during the approximately 3 m of shuttlecock–racket contact window, the racket’s sweep angular velocity, centripetal acceleration, and centripetal force all peaked one frame at the impact (frame 1) and then declined as shuttlecock speed increased, indicating substantial energy transfer from the racket to the shuttlecock at contact onset (Ljung et al., 1999). Within the first 1 m of contact, impact force reached its peak, with the primary component along the x-axis (anteroposterior direction), and the shuttlecock’s linear acceleration also peaked (∼48,520 m/s^2^). Hand sweep angular velocity and centripetal acceleration remained approximately constant throughout the contact window, whereas the forearm showed a slight early rise followed by a decline after shuttlecock-racket separation. The classic proximal-to-distal kinetic chain features a proximal peak followed by deceleration and a later distal peak (Wagner et al., 2014); although our window covers only the contact phase and not the full acceleration phase, the observed “racket deceleration during contact with a concomitant surge in shuttlecock speed” is consistent with the latter half of the chain, wherein energy transfer to the distal segment and the external object (the shuttlecock) is essentially completed by contact onset (Ramasamy et al., 2023). During contact, the distal segment (racket) undergoes marked velocity loss due to collision, while the proximal segment (hand and forearm) maintains or slightly increases sweep angular velocity and constrains the system via elevated centripetal force (Table 2 and 3); functionally, the hand-forearm complex tends toward stiffening to stabilize the racket face and facilitate impulse transmission (Frederic, 2005). Prior studies likewise indicate that shoulder internal rotation is key to higher smash speeds, with proximal segment motion explaining approximately 43.7% of the variance in shuttlecock speed (King et al., 2020). Skilled players produce faster smashes (Phomsoupha and Laffaye, 2014), and the elbow nearly stops just before impact (Salim et al., 2010); an isokinetic study further shows greater shoulder internal rotation strength in skilled badminton athletes (Ng and Lam, 2002); studies have shown that forearm motion exhibits an overall increasing trend throughout the entire stroke, which is consistent with the trend observed in our results (Shan et al., 2015). In addition, the relatively large standard deviations observed in Tables 2, 3 may be attributable to differences in technique. These findings align closely with our contact-window observation of “distal stabilization with proximal maintenance of angular velocity”.

The shoulder joint was adopted as a unified reference frame because, at a 1,000 Hz sampling rate, measurement noise and soft-tissue artifact amplify errors and instability in relative joint angular velocities. A shoulder-referenced representation is less data-demanding, more numerically stable, and allows all segments to be examined on a common time base for proximal-distal sequencing (Lees, 2002). Although current practice often relies on musculoskeletal models (e.g., Visual3D, OpenSim) to estimate joint forces and moments, the noise associated with high sampling rates degrades the stability of inverse dynamics solutions, yet high-speed swings necessitate such high sampling in the first place (Ramasamy et al., 2024; Tajik et al., 2025). Accordingly, we prioritized establishing millisecond-scale kinetic chain timing at the kinematic level and used it to provide stable, reliable loading inputs to the finite element analysis, thereby addressing the limitations of conventional musculoskeletal models in characterizing soft-tissue stress-strain.

A complete forehand smash typically involves shoulder internal rotation, elbow extension, and forearm pronation; however, because human reaction time exceeds 100 m (Kosinski, 2008), it is not possible to respond to millisecond-scale collision dynamics. Accordingly, we simplified the finite element model to focus on the tendons’ eccentric loading responses to contact-induced perturbations. Existing work has largely emphasized muscle modeling and simulations of active contraction under fixed loads (Oomens et al., 2003; Hedenstierna et al., 2008). Using the explicit dynamics module of finite element analysis, the present study is the first to simulate the instant of shuttlecock-racket contact in a badminton forehand smash. Quantitatively, the lack of directly comparable badminton studies necessitated comparison with related work, such as stress analyses of the radius and ulna during tennis strokes. Under a 300 N load at an elbow flexion angle of approximately 25°, maximum principal stresses of 565.26 MPa in the ulna and 172.11 MPa in the radius (Chen et al., 2023). In our badminton model, the corresponding values were markedly lower (ulna: 3.07 MPa; radius: 8.57 MPa). However, no finite element analyses of soft tissues during badminton strokes are currently available (Allen et al., 2009), limiting the extent of direct validation and cross-sport comparison. Beyond these joint- and bone-level quantities, our model further provides a tendon-level description of stress-strain behavior within the 3 m contact window.

**FIGURE 4:**
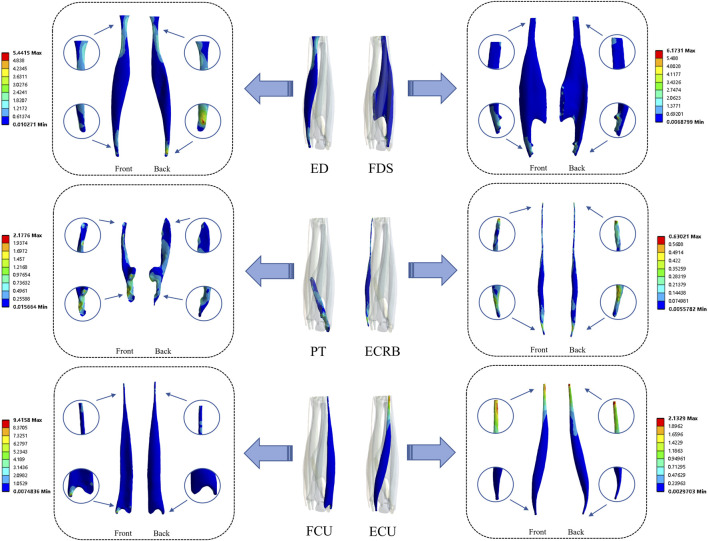
Stress distribution of the six muscles. Units: von Mises equivalent stress, MPa. EDC, extensor digitorum communis; FDS, flexor digitorum superficialis; PT, pronator teres; ECRB, extensor carpi radialis brevis; FCU, flexor carpi ulnaris; ECU, extensor carpi ulnaris.

As shown in Table 5 and Figures 3A, 4, the tendons did not share the collision load uniformly but exhibited distinct patterns of von Mises stress and strain over time. The flexor digitorum superficialis (0.37 MPa) and flexor carpi ulnaris (0.26 MPa) showed relatively high mean and peak stresses and strains, consistent with the smash phase’s need for sustained grip and ulnar-sided support (Frederic, 2005; Ramasamy et al., 2022). However, the extensor digitorum communis actually displayed the highest stress among all tendons (average 0.63 MPa, peak 0.75 MPa), while its strain remained in the mid-range. In contrast, flexor digitorum superficialis exhibited the largest equivalent strain (average 1.65E-03), indicating that it plays a primary role in absorbing impact energy and maintaining racket-handle coupling. Notably, the peak tendon strain was very small (maximum von Mises equivalent strain 1.83 × 10^−3^; Table 5), indicating a small-strain regime well below the typical nonlinear (“toe”) strain range reported for tendons and supporting the use of a linear-elastic approximation in the present millisecond-scale simulations (Wang, 2006). Together, the combination of high extensor digitorum communis and pronator teres (0.33 MPa) stresses with high flexor digitorum superficialis strain suggests a division of labor in which extensor digitorum communis and pronator teres chiefly “hold” the forearm-hand complex against perturbation, whereas flexor digitorum superficialis deforms more to buffer the collision. The time-series data further clarify this coordination. From contact onset, pronator teres and flexor digitorum superficialis stresses rose steeply and reached their maxima at approximately 60%–80% of the contact window, whereas extensor digitorum communis stress continued increasing and peaked closer to the end of contact (Figure 3A). This temporal pattern mirrors a “pronation-driven, co-contraction-stabilized” strategy: pronator teres provides strong pronation to resist the shuttlecock-induced tendency toward supination (Figure 3A), while concurrent flexor digitorum superficialis-extensor digitorum communis co-contraction stiffens the metacarpophalangeal region and enhances overall forearm-hand rigidity (Frederic, 2005; Hoshika et al., 2020). By contrast, extensor carpi radialis brevis and the ulnar-side wrist tendons showed comparatively lower stresses and small strains, implying a more supportive, stabilizing role and minimal reliance on large-amplitude pure wrist flexion or radial/ulnar deviation during impact. When these tendon-level findings are combined with the kinetic data in Tables 2, 3 – racket sweep angular velocity peaking before contact, hand sweep angular velocity remaining approximately constant, and forearm angular velocity rising slightly and then declining after separation–the emerging picture is one of distal “stiffening plus pronation maintenance” during the millisecond-scale collision. In other words, the latter half of the macroscopic kinetic chain (Wagner et al., 2014; Zhang et al., 2016) is implemented mechanically by strong pronator teres-driven pronation, superimposed on flexor digitorum superficialis-extensor digitorum communis co-loading that increases dynamic stiffness and maintains racket-face orientation rather than by large wrist excursions.

In damage mechanics, macro- and micro-scale experiments alike have shown that determining the tendon stress-strain curve via *in vitro* tensile testing constitutes a fundamental paradigm in biomechanics (Yang et al., 2013). As load increases, tendons operating in the nonlinear region begin to incur damage (Wang et al., 2021). As indicated in Table 5 and Figure 4, compared with the flexor digitorum superficialis and pronator teres, the extensor digitorum communis experiences the highest stress. Coupled with the large but still sub-failure strains seen in flexor digitorum superficialis, this suggests that imbalance between the forearm extensor and flexor groups may increase the risk of sports injury and degrade performance (Kamalden et al., 2021), implying that badminton athletes should strengthen the extensors. Moreover, whether in training or competition, repetitive strokes induce fatigue, which reduces shot accuracy and effectiveness (Zhang, 2020) and increases the likelihood of injury (Richardson, 1983). Studies in sports such as tennis and cricket indicate that repeated supination and pronation may exacerbate the risk of tendinopathy (Nayak et al., 2010). Although our finite element results indicate that, during the forehand smash, forearm muscle loading lies within the linear region of the stress-strain response, repetitive loading has been shown to induce tendon microdamage leading to failure (Wren et al., 2003), and the proportion of overuse injuries in badminton is three times that of traumatic injuries (Miyake et al., 2016).

Several limitations should be considered when interpreting these results. Shuttlecock–racket contact lasts only a few milliseconds; at 1,000 Hz this yields ∼3–4 frames. Thus, normalizing the contact window (100 points) and applying polynomial smoothing are used for between-trial comparison but do not increase the true temporal resolution and may affect apparent peak timing and curve shape. Accordingly, our inferences rely mainly on peak magnitude outcomes (e.g., maximum resultant force and peak tendon stress/strain) rather than detailed within-contact temporal profiles. In addition, direct comparison and validation against badminton-specific soft-tissue finite element studies are limited, and some kinematic definitions are not directly comparable with prior reports. Displacement-based verification showed non-negligible errors in certain cases, which may propagate to estimated tissue responses. Finally, the modelling necessarily involves assumptions in geometry, material properties and boundary/loading conditions, which may influence absolute stress–strain values. Moreover, our musculotendon architecture was adopted from a generic open-source model (not subject-specific MRI), which may limit the generalizability of the predicted stress/strain magnitudes.

## 5 Conclusion

This study coupled 1,000 Hz motion capture with explicit finite element modeling to resolve millisecond-scale shuttlecock-racket contact mechanics and tendon loading in the badminton forehand smash. The results indicate a non-uniform tendon load distribution, with relatively higher loading in the extensor digitorum communis and pronator teres and evidence of distal stabilization via wrist flexor–extensor co-loading during impact. Although the predicted stress/strain magnitudes are well below acute failure thresholds, these loading patterns may be relevant to cumulative overuse risk under repetitive exposure and may help inform targeted strengthening and conditioning of the wrist extensors and the pronation chain.

## Data Availability

The raw data supporting the conclusions of this article will be made available by the authors, without undue reservation.
